# Glimmers of hope on the Ebola front

**DOI:** 10.2471/BLT.14.031014

**Published:** 2014-10-01

## Abstract

Daniel Bausch has been assisting with patient care during the current Ebola virus disease outbreak in western Africa and – as part of a WHO-led international collaboration – is exploring the possible use of experimental therapies and vaccines. He tells Fiona Fleck why this outbreak is different.

Q: Last month you paid tribute to your colleague Dr Sheikh Humarr Khan from Sierra Leone, who ran the only medical unit in the world devoted exclusively to the care of patients with viral haemorrhagic fever and who died of Ebola virus disease in July. Why are so many health workers dying, even those wearing the recommended protective clothing?

A: There is a lot of focus on personal protective equipment – the suits, gowns and masks seen on so many photographs of the outbreak. But this is only one of many important aspects of safe patient care. In many places the demand for patient care has outstripped the available human resources. For example, a couple of months ago a WHO physician, David Brett-Major, and I made rounds at the Kenema treatment centre in Sierra Leone, where the nurses were on strike. The two of us were the only health-care workers on that ward of 60 patients with Ebola virus disease. Even if you wear the recommended gear, much more is needed, such as supervision and sanitation officers to decontaminate the area regularly. Asking a health-care worker to safely care for patients without support personnel is like asking a pilot to fly a plane without mechanics and flight controllers.

Q: Thousands more cases are expected in the coming weeks, but with so many health workers who are sick or have died, the health workforce in the affected countries is being depleted. How can WHO and its partners recruit more staff quickly enough to support these countries on the front line of this outbreak?

A: First you need to find the right people. Ideally they should have expertise in viral haemorrhagic disease, which – before this outbreak – was rare. Very few of these experts were clinicians doing patient care. Most were laboratory researchers and epidemiologists. Recruiting new people is not easy. Many of them have jobs they cannot leave and families that don’t want them to go. It’s just as difficult to recruit people in the affected countries, especially when the previous post holder died, and no one in western Africa is getting rich from doing this kind of patient care. We, the expatriate field workers, get a lot of media attention, but – when you think of the risks they take – the real heroes in this outbreak are the Guineans, Liberians and Sierra Leoneans who are living and working in their communities, and facing unimaginable fatigue and stress every day. We go for a month, then get a rest. For them it never stops.

*Q: You and your colleagues published a call for action to promote more research on the prevention and treatment of Ebola virus and other filovirus diseases in *The Journal of Infectious Diseases* in 2007* (*196:S136–41). What came of this?*

A: We called for the establishment of infrastructure for clinical research on filoviruses in Africa, with skilled staff and a legal–logistical framework in the affected countries and internationally. We proposed that research protocols, data collection forms and culturally-appropriate methods of gaining informed consent should be prepared and approved in advance of any outbreak through an international ethics review process. If these and other basic principles had been put in place back then – I’m not saying we could have prevented the current outbreak – we would have been in a better position to tackle it. We proposed a way forward. It was a missed opportunity. Now people are discussing this again.

Q: In the 2007 article, you noted that, in the absence of vaccines and drugs, quarantining and contact tracing were the key public health approaches to stopping a filovirus outbreak. Why have these failed in some places during the current outbreak?

A: It’s not the first outbreak where local communities have resisted these measures, but it’s the one where resistance has been the most intense and violent. It’s easy to point a finger and say “how stupid” that people in the affected communities don’t follow the measures we recommend. But we have to understand that this distrust of foreigners is in part the legacy of the unjust colonial era and less than ideal governance since.

Q: WHO has faced a storm of criticism over its response to the outbreak. Could the crisis have been prevented and, if so, at what point?

A: When I went to Guinea in April, shortly after the epidemic was declared, it seemed to me to be “a routine Ebola virus disease outbreak”. The days started with the usual meeting at the WHO Representative’s office to go over the epidemiological updates with the usual international partners: Médecins Sans Frontières (MSF), CDC and the Red Cross. At the beginning, people say: “We know how to do this, let’s dive in and get this mopped up.” When I came home at the end of May, it looked as if the outbreak was coming to an end, with very few new cases. The WHO clinical team debated whether we needed to recruit more clinicians. But you can’t monitor every village. You watch and wait. Sometimes it’s over. Sometimes someone knocks on the door again saying: “Six people recently died in a village.” And that’s what happened. When I went to Sierra Leone six weeks later, there was a distinctly different feel about the outbreak response. The first morning I went to the WHO Representative’s office, there were very few members of staff from WHO and the other international organizations. Clearly they had limited human and financial resources, and had expended them.

Q: Is that when you realized the outbreak was not getting under control?

A: Yes. We clearly recognized the need for more clinicians, logisticians, epidemiologists, everything. MSF said they had the capacity to establish a ward in Kailahun but not in Kenema – the two worst affected districts in Sierra Leone. It was the first time since MSF had started working on Ebola virus disease outbreak response that I heard them say they didn’t have enough resources. After a few months everyone is exhausted and needs to go home. When clinicians get tired, their work becomes more dangerous, but it’s expensive to replace them. The outbreak response had outstripped the available resources. WHO recognized this and sounded the alarm at a meeting in Accra, Ghana (2–3 July). Since then, various groups have pledged resources. For example, CDC has deployed more than 50 epidemiologists across the affected region and WHO has increased their personnel as well, but we are all late and it has gotten out of control. It’s too simplistic to lay the blame on one group. There has been a lot of finger pointing at WHO, no one is immune to criticism, but WHO has suffered a loss of personnel and resources. So it’s not only about what we should have done at any particular time, but the whole foundation for an international public health response that has been eroded by the global economic downturn.

Q: Why was this Ebola virus disease outbreak different from previous ones?

A: It started in the border area of the three countries: remote, impoverished areas with virtually no health or surveillance infrastructure. So it has been difficult to do contact tracing, organize isolation and provide treatment. The local populations regularly cross the borders to visit or trade with their ethnic kin. The borders are porous, but the public health authorities have no system for coordinating cross-border matters and, even within each country, health systems are weak at best. This has been exacerbated by language barriers: in Guinea the national language is French, in Liberia and Sierra Leone it is English.

“The whole foundation for an international public health response … has been eroded by the global economic downturn.”

Q: Research is vital for learning about Ebola virus disease and developing disease control methods. What have been the challenges for research in this outbreak?

A: It’s not easy to find people to work in the outbreak let alone those who are able to do research. For example, during the war when the medical school was shut down, Liberia had only a handful of trained doctors in the whole country. Over a decade ago, WHO, Tulane, and other partners started the Mano River Union Lassa Fever Network to build capacity for combatting that disease (another viral haemorrhagic fever) in Sierra Leone, Guinea and Liberia. I remember trying to recruit a new laboratory technician looking at résumés from people who had spent two or three years in refugee camps and who hadn’t had the opportunity to develop skills in the sciences. You can give them the technical training, but that’s not enough for them to become a principle investigator, who can frame a hypothesis, write a research proposal, seek funds, implement the project with a team, analyse the data and publish the results. This requires a culture of research and this is something we were trying to build. But so many health-care workers have died of Ebola virus disease, in addition to the personal tragedy, this loss is a major blow to these efforts to advance research in these countries.

Q: You were at the WHO meeting last month to discuss the possible use of experimental vaccines and drugs, has this brought the end of the outbreak closer? 

A: WHO brought together a vast array of experts from regulatory agencies, pharmaceutical companies, public health agencies and scientific institutes. The meeting was productive but the devil is in the detail. We have a moral imperative to accelerate the pace of these experimental products through the pipeline, but the challenge before us is formidable and the path not 100% clear. At present, in the absence of vaccines and therapies, many who are sick don’t want to be traced, but prefer to disappear into the forest or urban jungle. If we can start providing vaccines and drugs, hopefully in the not too distant future, the problem will change and people will start knocking on the door demanding prevention and treatment, so this is a public health strategy as well. But we won’t be able to get experimental compounds to everyone in need. Stemming this outbreak will still depend primarily on the classic strategy of case identification, with isolation and treatment, and contact tracing.

Q: Are there other glimmers of hope?

A: We have a major public health crisis across this region that threatens to get larger. There are still measures we can take to prevent the crisis spreading and we must continue these efforts. We are working to make things happen faster, but this requires cooperation from many groups across the world. Everyone recognizes the need to take action to stop the outbreak, but it’s not easy. I do think that everyone is heeding the call now and doing what they can. In the long-term, the scale and public profile of this outbreak means that potential vaccines and therapies that were stalled are now being pushed through clinical trials. I hope that we have something to offer people during this outbreak but, at the very least, we must emerge more prepared for the next. Another long-term glimmer of hope is that there are discussions about how to help these three long-suffering countries. They are going to need a Marshall Plan for reconstruction when this is over, with infrastructure and educational programmes to put some of the world’s poorest nations on the road to health and prosperity.

